# Clinical Utility of Polymerase Chain Reaction (PCR) in Diagnosing and Managing Bacterial Infections: A Case Series of Three Patients With Acinetobacter baumannii

**DOI:** 10.7759/cureus.80823

**Published:** 2025-03-19

**Authors:** Jalal Ibrahim, Riley J Stone, Torrin Jacobsen, Robert Norman

**Affiliations:** 1 Medicine, Lake Erie College of Osteopathic Medicine, Bradenton, USA; 2 Anthropology, University of South Florida, Tampa, USA; 3 Dermatology, Nova Southeastern University Dr. Kiran C. Patel College of Osteopathic Medicine, Tampa, USA

**Keywords:** abscess, acinetobacter baumannii, antibiotic sensitivity and resistance, bacterial infection, bacterial rash, pcr test

## Abstract

This case series highlights the critical role of polymerase chain reaction (PCR) in diagnosing and managing bacterial infections in dermatology. We present three cases of patients with the same bacterial infection, identified through PCR, and discuss the implications for treatment. The series highlights the advantages of PCR over traditional culture methods, emphasizing its ability to deliver rapid, accurate diagnoses and facilitate targeted therapeutic interventions. Unlike cultures, which can be time-consuming, PCR provides a quicker and more precise identification of pathogens. Additionally, we outline the cost-saving potential of PCR, as its efficiency in pinpointing infections can reduce the need for broad-spectrum antibiotics, minimize unnecessary diagnostic procedures, and shorten the duration of patient illness, thereby contributing to overall healthcare savings.

## Introduction

*Acinetobacter baumannii* is an opportunistic pathogen that has been greatly increasing in prominence over the years and is known to cause a variety of severe infections in the healthcare setting [1]. This pathogen can cause meningitis, bacteremia, endocarditis, skin and soft tissue infections, urinary tract infections, and pneumonia [1]. These types of infections are already a cause for concern, but when paired with their ability to thrive in healthcare environments - particularly in military medical facilities and intensive care units - this pathogen presents an even greater cause for concern [1]. Patients who are immunocompromised or experience prolonged hospital stays face especially heightened risks from *A. baumannii* infections due to their vulnerability [2]. Moreover, the clinical management of *A. baumannii* is further complicated by its known ability to create adaptable biofilms and its multi-drug resistance characteristics [2]. This multi-drug resistance leaves limited treatment options and increases the necessity for fast and accurate diagnosis.

Traditional diagnostic methods, such as bacterial cultures, although reliable, lack the turnaround time and sensitivity needed for early detection of *A. baumannii*. This is because these organisms are often difficult to de-stain [3]. Since there is no definitive metabolic test for this pathogen, it is challenging to distinguish it from other non-fermenting Gram-negative bacteria [3]. Thus, there is an increased necessity for diagnostic techniques that rapidly and accurately identify pathogens. Polymerase chain reaction (PCR) addresses these limitations by providing rapid, precise, and detailed pathogen identifications within a four- to six-hour period [4]. Although this may be more expensive upfront compared to traditional cultures, which typically cost around $200 per test versus $300, PCR tests can still potentially reduce overall healthcare costs [5]. This reduction comes from the shorter diagnostic process, minimization of unnecessary or inefficient treatments, and decreased hospital stay durations.

We present a case series of three patients diagnosed with high concentrations of *A. baumannii*, where PCR was performed as the diagnostic tool. By focusing on *A. baumannii*, a pathogen with high clinical relevance, the goal is to highlight the use of PCR as a diagnostic tool in cases of complex bacterial infections. In these cases, the PCR results influenced differing treatment plans due to the identification of different combinations of bacteria.

## Case presentation

This case series includes three patients who tested positive for *A. baumannii* via PCR.

Case 1

Patient 1, a 47-year-old female, presented to the dermatology clinic with concerns regarding biopsy results, which were done to rule out any malignancy and discomfort, as well as an abscess in the groin area. The patient experienced moderate pain when the abscess came into contact with jewelry and clothing. The biopsy sites, located on the left inferior and superior neck and the right anterior and posterior neck, were healing appropriately. The initial clinical diagnoses for this patient included hidradenitis suppurativa, fibroepithelial polyp, xerosis of the skin, and a bacterial infection. PCR testing identified high levels of *A. baumannii* and *Klebsiella pneumoniae*, as well as medium levels of *Corynebacterium* species and *Staphylococcus* species.

The patient also reported itching and pain around the right groin, which progressed to a larger boil. This led to the drainage of the abscess and the use of PCR to identify the specific bacterial pathogens responsible for the patient's symptoms. Treatment with levofloxacin was initiated, helping to resolve the signs and symptoms.

PCR results were instrumental in identifying bacterial infections, assessing antibiotic resistance profiles, and guiding therapeutic decisions. The PCR analysis provided insights into the resistance genes expressed by the bacteria, enabling clinicians to select appropriate antibiotics and avoid ineffective or undesirable treatments. Additionally, the PCR results quantified the bacterial load and identified genetic characteristics, including potential treatment resistance, thus offering a range of pharmaceutical options.

For this patient, the PCR results indicated that potential treatments included levofloxacin, ciprofloxacin, or meropenem (Table 1). This information allowed the dermatologist to choose the most suitable antibiotic based on the patient's preference and clinical considerations.

**Table 1:** The chart shows the bacteria detected in three different patient cases, along with the antibiotics suggested and those actually given, as well as the outcome. It highlights the challenge of treating resistant bacteria like *Acinetobacter baumannii* and the importance of adjusting treatments based on how the patient responds. TMP-SMX: Trimethoprim-Sulfamethoxazole

PCR detection in microbial organisms and antibiotic suggestions			
Bacteria detected	Case 1	Case 2	Case 3
	High levels of *Acinetobacter baumannii* and *Klebsiella pneumoniae*, with medium levels of *Corynebacterium* species and *Staphylococcus* species	High levels of *Acinetobacter baumannii* and *Cutibacterium acnes*, with moderate levels of *Enterococcus faecalis*.	High levels of *Acinetobacter baumannii* with moderate levels of *Enterococcus faecalis*.
Antibiotics suggested	Levofloxacin, Ciprofloxacin, Meropenem	Levofloxacin, Amoxicillin/Clavulanic acid, Minocycline, and Bactrim (TMP-SMX)	Levofloxacin, Amoxicillin/Clavulanic acid, Ciprofloxacin, Doxycycline, Bactrim, Meropenem
Antibiotic given	Levofloxacin	Amoxicillin/Clavulanic acid	Doxycycline
Outcome	Improvement	Improvement	Improvement

Case 2

Patient 2, a 61-year-old female, presented to the dermatology clinic for a follow-up of a persistent rash that was constantly itching, and she believed it to be intertrigo. The patient also reported the presence of sores/rash on the scalp and a lesion on the back, characterized by erythematous, flaky, and moist, crusted areas with blue pigmented spots. The symptoms, which have been ongoing for three months, are notably itchy, burning, and tender.

Given the persistence of the condition, a PCR test was ordered to assess for a possible bacterial infection. While awaiting the PCR results, the patient was prescribed cephalexin. Once the patient returned for her follow-up appointment to receive the PCR results, she was switched to amoxicillin/clavulanic acid, and her condition improved. 

The PCR results indicate the presence of high levels of *A. baumannii*, high levels of *Cutibacterium acnes*, and moderate levels of *Enterococcus faecalis*. Based on these results, and considering the patient's medical history along with the specific strain and resistance profile of *A. baumannii*, the following medications have been recommended: amoxicillin/clavulanic acid, levofloxacin, minocycline, and trimethoprim-sulfamethoxazole (Table 1).

This information will guide the dermatologist in selecting the most effective treatment regimen for the bacterial infection contributing to the patient's symptoms. Additionally, the PCR helps gauge the approximate pricing for a typical course of treatment for *A. baumannii*, which can make the patient more compliant in treating her condition effectively.

Case 3

Patient 3, a 72-year-old female, presented to the dermatology clinic with a persistent and worsening rash in the genital area that had been ongoing since November 2023. The rash had since spread to other parts of her body, prompting her visit. The patient also reported significant itchiness, which intensified after exposure to the beach.

Initial evaluation suggested a potential bacterial infection, leading to the decision to perform a PCR test to identify the causative microorganism. In the interim, the patient was prescribed doxycycline as a prophylactic measure. She is scheduled to return in two weeks for a follow-up and to review the test results.

The PCR results revealed high levels of *A. baumannii* and moderate levels of *E. faecalis*. The test results also provided insights into possible resistance genes present in the bacteria, recommended treatment regimens similar to those indicated for Case 1 and Case 2, and detailed information about the pricing of the medications. By obtaining much of the needed information upfront, it decreases the likelihood of needing a trial-and-error approach. A summary of all three cases is provided in Table 1 to give a clear layout of what occurred.

## Discussion

The use of PCR as a diagnostic tool has proven crucial in identifying specific bacterial strains, assessing antibiotic resistance profiles, and guiding appropriate treatment strategies for patients. In all three cases presented, PCR results were available within approximately six hours, compared to traditional methods, such as bacterial cultures, which would have required significantly more time, although they offer comparable accuracy [4,6,7]. This rapid turnaround allows clinicians to formulate treatment plans more promptly.

The PCR results in these cases had a direct impact on treatment decisions by providing detailed information on bacterial load and resistance profiles. In Case 1, the detection of *A. baumannii* and co-infection with *K. pneumoniae* led to the choice of levofloxacin due to its effectiveness against Gram-negative bacteria [8]. In Case 2, the identification of *A. baumannii*, along with *C. acnes* and *E. faecalis*, facilitated a more informed selection of antibiotics. Similarly, in Case 3, the PCR results offered several potential treatment options for managing the infection.

The primary benefit of PCR in these cases lies in its rapid and accurate diagnostic capabilities, which are particularly critical for managing multi-drug-resistant infections. Delays in diagnosis and treatment can exacerbate patient outcomes, especially in hospital settings where *A. baumannii* is recognized as a significant concern due to its prevalence and resistance patterns [9]. Additionally, prolonged hospital stays caused by diagnostic delays only serve to contribute to higher healthcare costs.

However, there are also potential implications associated with the use of PCR. Its high sensitivity enables the detection of very low levels of bacteria, which may lead to concerns about overtreatment. Literature on this topic suggests that such concerns may be overstated. For instance, a study comparing PCR to direct immunofluorescence (DIF) for respiratory viruses found no significant change in treatment plans [10]. Similarly, another study demonstrated that rapid influenza PCR in the emergency department actually resulted in reduced antibiotic use and facilitated more timely antiviral therapy [11].

## Conclusions

In summary, this case series underscores the value of PCR in diagnosing complex bacterial infections. The rapid and precise identification of pathogens and their resistance profiles significantly enhances patient management and treatment outcomes. Despite challenges such as testing costs and accessibility in resource-limited settings, the benefits of PCR technology are substantial. In clinical practice, PCR can contribute to more targeted treatment strategies and reduce the risk of over-treatment. PCR testing can always be used in situations like these case presentations; whether there is an absolute need for it is up to the provider to determine for accurate results. 
